# The transvaginal hybrid NOTES versus conventionally assisted laparoscopic sigmoid resection for diverticular disease (TRANSVERSAL) trial: study protocol for a randomized controlled trial

**DOI:** 10.1186/1745-6215-15-454

**Published:** 2014-11-20

**Authors:** Jonas D Senft, Rene Warschkow, Markus K Diener, Ignazio Tarantino, Daniel C Steinemann, Sebastian Lamm, Thomas Simon, Andreas Zerz, Beat P Müller-Stich, Georg R Linke

**Affiliations:** Department of General, Abdominal and Transplantation Surgery, University of Heidelberg, Im Neuenheimer Feld 110, 69120 Heidelberg, Germany; Institute of Medical Biometry and Informatics, University of Heidelberg, Im Neuenheimer Feld 305, 69120 Heidelberg, Germany; Study Center of the German Surgical Society (SDGC), University of Heidelberg, Im Neuenheimer Feld 110, 69120 Heidelberg, Germany; Department of Surgery, Kantonsspital Baselland, CH - 4101 Bruderholz, Switzerland; Department of Surgery, GRN Klinik, Alte Waibstadter Straße 2, 74889 Sinsheim, Germany

**Keywords:** Diverticular disease, Laparoscopy, Natural orifice transluminal endoscopic surgery, NOTES, Randomized controlled trial, Sigmoid resection, Study protocol, Transvaginal

## Abstract

**Background:**

Natural orifice transluminal endoscopic surgery (NOTES) is the consequence of further development of minimally invasive surgery to reduce abdominal incisions and surgical trauma. The potential benefits are expected to be less postoperative pain, faster convalescence, and reduced risk for incisional hernias and wound infections compared to conventional methods. Recent clinical studies have demonstrated the feasibility and safety of transvaginal NOTES, and transvaginal access is currently the most frequent clinically applied route for NOTES procedures. However, despite increasing clinical application, no firm clinical evidence is available for objective assessment of the potential benefits and risks of transvaginal NOTES compared to the current surgical standard.

**Methods:**

The TRANSVERSAL trial is designed as a randomized controlled trial to compare transvaginal hybrid NOTES and laparoscopic-assisted sigmoid resection. Female patients referred to elective sigmoid resection due to complicated or reoccurring diverticulitis of the sigmoid colon are considered eligible. The primary endpoint will be pain intensity during mobilization 24 hours postoperatively as measured by the blinded patient and blinded assessor on a visual analogue scale (VAS). Secondary outcomes include daily pain intensity and analgesic use, patient mobility, intraoperative complications, morbidity, length of stay, quality of life, and sexual function. Follow-up visits are scheduled 3, 12, and 36 months after surgery. A total sample size of 58 patients was determined for the analysis of the primary endpoint. The confirmatory analysis will be performed based on the intention-to-treat (ITT) principle.

**Discussion:**

The TRANSVERSAL trial is the first study to compare transvaginal hybrid NOTES and conventionally assisted laparoscopic surgery for colonic resection in a randomized controlled setting. The results of the TRANSVERSAL trial will allow objective assessment of the potential benefits and risks of NOTES compared to the current surgical standard for sigmoid resection.

**Trial registration:**

The trial protocol was registered in the German Clinical Trials Register ( DRKS00005995) on March 27, 2014.

**Electronic supplementary material:**

The online version of this article (doi:10.1186/1745-6215-15-454) contains supplementary material, which is available to authorized users.

## Background

### Rationale

Since natural orifice transluminal endoscopic surgery (NOTES) was described by Kalloo *et al.* it has gained the attention of surgeons and gastroenterologists worldwide [1]. Aiming to further minimize surgical trauma by reducing transabdominal incisions, NOTES may be seen as an attempt to further develop minimally invasive surgery. The potential benefits of avoiding abdominal incisions with NOTES are less postoperative pain, faster convalescence, and reduced risk of incisional hernias and wound infections. However, these potential advantages have not yet been proven conclusively.

Several NOTES concepts are currently in development. Though pure NOTES is performed exclusively via transluminal access, hybrid NOTES combines transluminal access with conventional laparoscopic access. Pure NOTES procedures are technically challenging and have been applied only in sporadic clinical cases [2–4]. In contrast, hybrid NOTES procedures allow the use of rigid instruments and are technically very similar to conventional laparoscopic procedures. Surgical access can be created and closed under laparoscopic view using a transabdominally placed trocar and the specimen removed via transluminal access. Due to these benefits, hybrid NOTES procedures have advanced to clinical application [5–9].

Based on gynecological experience, transvaginal access was the first transluminal access and advanced to the most frequently applied route for NOTES [7]. Several studies have confirmed the feasibility and safety of transvaginal cholecystectomy [5, 6, 8] and colonic resection [10, 11] using the hybrid NOTES approach.

NOTES is still met with broad skepticism because its potential benefits are generally doubted and potential access-related complications feared. However, despite increasing clinical application, there is no firm evidence for objective assessment of the potential benefits and risks of NOTES. In particular, no study has compared transvaginal hybrid NOTES sigmoid resection and conventional laparoscopic surgery in a randomized controlled setting.

### Objective

The TRANSVERSAL (‘Transvaginal hybrid NOTES versus conventionally assisted laparoscopic sigmoid resection for diverticular disease’) trial is a randomized controlled trial intended to evaluate transvaginal hybrid NOTES and conventionally assisted laparoscopic sigmoid resection. The study design allows objective assessment of the potential benefits and risks of transvaginal NOTES compared to the current laparoscopic standard for sigmoid resection.

## Methods/Design

### Trial design

**Figure 1:**
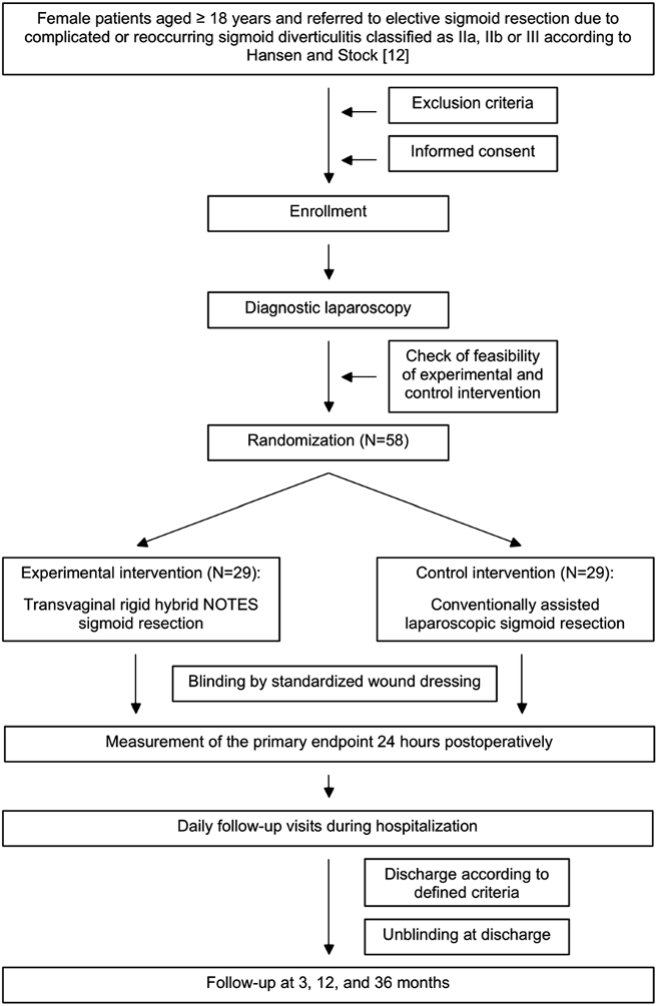
**Trial scheme.**

The TRANSVERSAL trial is a randomized controlled multicenter trial comparing transvaginal hybrid NOTES sigmoid resection (experimental intervention) to conventionally assisted laparoscopic sigmoid resection (control intervention) and is designed as a parallel group superiority trial. The trial scheme is illustrated in Figure 1.

### Study population and eligibility criteria

All female patients aged ≥18 years and referred to elective sigmoid resection due to diverticulitis of the sigmoid colon will be screened for inclusion (Table 1). Patient screening comprises an investigation of the patient’s medical history and findings, a physical examination, and a routine gynecological examination performed by a gynecologist in all cases.

**Table 1:** **Inclusion and exclusion criteria**

Inclusion criteria	Exclusion criteria
• Female patients indicated for elective sigmoid resection due to at least two episodes of uncomplicated diverticulitis or first episode of complicated diverticulitis classified as IIa or IIb according to Hansen and Stock [12]	• ASA classification higher than III
• Age ≥18 years	• Inability to consent
• Informed consent	• Pregnancy
	• Genital infections
	• Neoplasms of vulva, vagina, or cervix
	• Douglas endometriosis
	• History of pelvic floor repair
	• Chronic inflammatory bowel disease
	• Fibromyalgia
	• Psychiatric disorder
	• Regular use of analgesics, steroids, or antidepressants

ASA, American Society of Anesthesiologists.

### Trial locations

The TRANSVERSAL trial will be conducted at three centers with expertise in transvaginal hybrid NOTES:

-  Department of General, Abdominal, and Transplantation Surgery, University of Heidelberg, Heidelberg, Germany
-  Department of Surgery, Kantonsspital Baselland, Bruderholz, Switzerland
-  Department of Surgery, GRN Klinik Sinsheim, Sinsheim, Germany

### Organizational structure and responsibilities

The principal investigator (B.M.) is primarily responsible for the preparation of the study protocol and the case report form (CRF), the organization of steering committee meetings, and the dissemination policy. At each participating center, a lead investigator (G.L., A.Z., T.S.) is responsible for screening, recruitment, data collection, and completion of the CRFs. All lead investigators are members of the study steering committee, which oversees study progress and safety. Prior to creation of the study protocol, standardization of the intervention, perioperative management, patient recruitment, and study visits was homogenized during steering committee meetings. Further meetings are intended to occur before enrollment of the first patient. Surgery will be performed by the principal investigator and the lead investigators with an experience of >50 laparoscopic colorectal resections and >20 transvaginal hybrid NOTES procedures. Further meetings are intended to occur before enrollment of the first patient. Patient enrollment, randomization, and data management will be carried out by the Study Center of the German Surgical Society (SDGC) at the University of Heidelberg.

### Sample size

A total of 58 patients will be randomized for this trial, 29 patients per group.

### Recruitment and trial timeline

Female patients aged ≥18 years presenting for elective sigmoid resection due to at least two episodes of uncomplicated diverticulitis or the first episode of complicated diverticulitis classified as IIa or IIb according to Hansen and Stock [12] will be further screened for eligibility criteria by surgical consultants at the participating centers. Surgical consultants from all centers will be informed about the TRANSVERSAL trial and its inclusion criteria during department meetings. For informed consent, the patients will be verbally introduced to the trial by a member of the research group and in writing via the Patient Information Sheet (Additional files 1, 2, 3 and 4). Informed consent will be obtained from each patient before inclusion in the study.

Before inclusion in the study, the feasibility of both techniques will be confirmed by diagnostic laparoscopy to avoid inclusion of patients who are immediately converted to open surgery. Potential reasons for conversion to open surgery are pronounced adhesions or tissue scarification due to prior surgery or prior episodes of diverticulitis. However, the decision is subject to the assessment of the respective surgeon. Therefore, the reasons for technical infeasibility and other reasons for exclusion will be recorded anonymously alongside descriptive data for all screened patients and presented in the final study report. If experimental or control interventions seem technically feasible to the surgeon during diagnostic laparoscopy, patients will be enrolled at the participating centers. A total of 100to 150 female patients are expected to be referred for elective surgery for diverticulitis. Based on the experiences in our previous study, we expect a recruitment rate of 60 to 70% [11]. Recruitment strategies, such as provisions or financial compensation, are not intended. The recruitment of 58 patients is planned to be finished within 24 months. The time interval from first patient in to last patient out will be 60 months.

### Randomization, allocation concealment, and blinding

**Figure 2:**
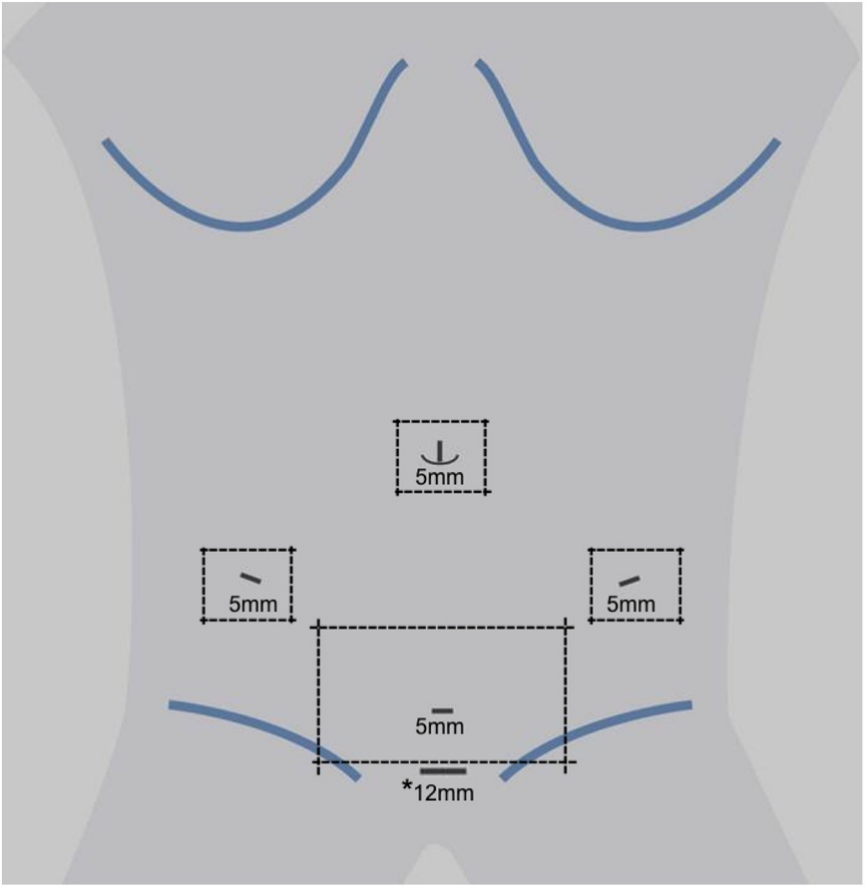
**Trocar positions and standardized wound dressing for the experimental intervention.** *transvaginal trocar.

**Figure 3:**
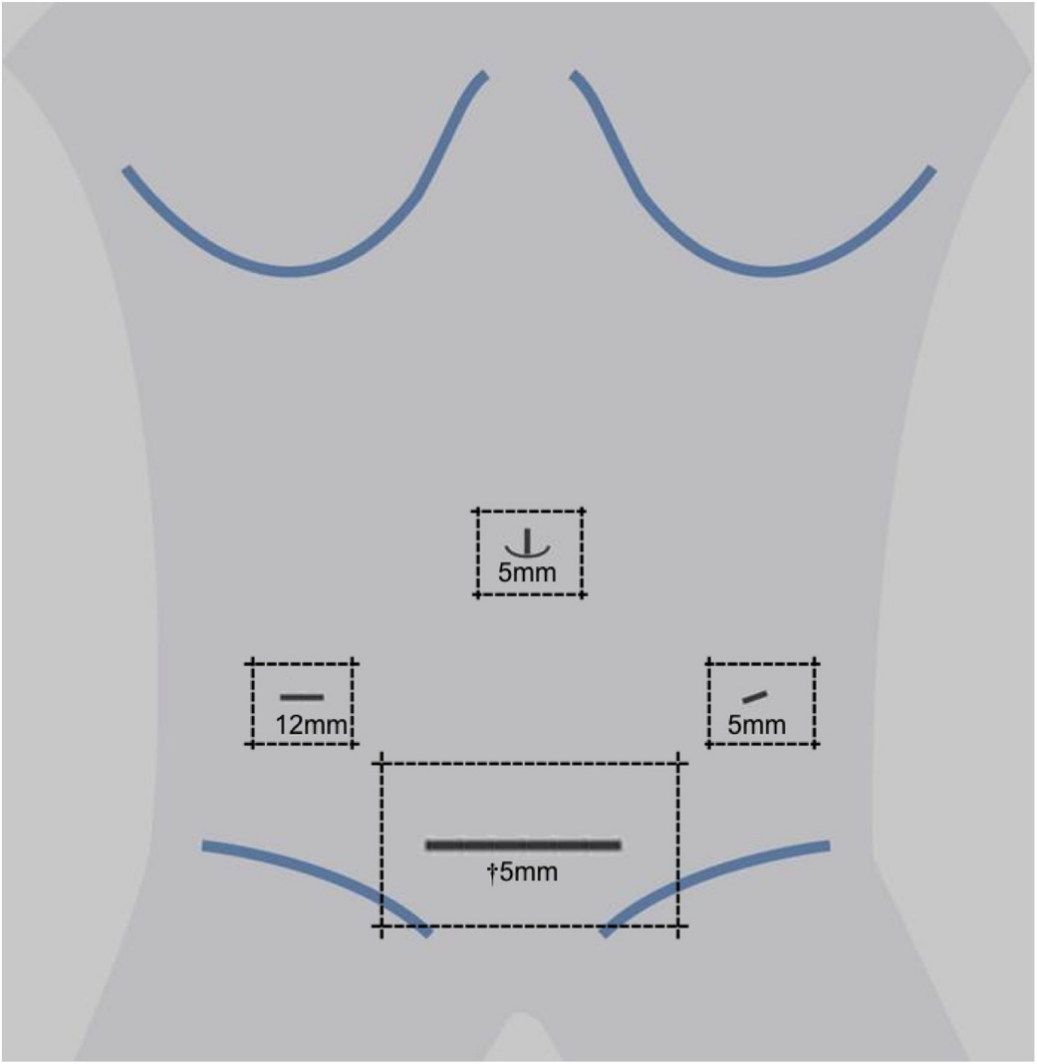
**Trocar positions and standardized wound dressing for the control intervention.** †a 5 mm trocar incision will be extended to Pfannenstiel minilaparotomy.

The SDGC will enroll and randomize patients immediately after diagnostic laparoscopy and confirmation of the technical feasibility of both procedures by the surgeon. The patients will be randomized using an online randomization tool ( http://www.randomizer.at). Randomization will be stratified by computer-generated permuted blocks of varying size with a 1:1 ratio to the experimental and control intervention groups. Patient allocation will be revealed to the surgeon after randomization by a study nurse using a written form, which will be destroyed immediately after unveiling. A standardized wound dressing will be applied after both techniques to cover the trocar and minilaparotomy sites (Figures 2 and 3). Investigators and patients will be blinded to the group affiliation until removal of the wound dressing at discharge. The study visits will be performed by investigators who are not involved in the surgical procedures. The wound dressing will be changed with the patient blinded using blinding glasses on postoperative day (POD) 2 and at 3-day intervals thereafter. At each center, a study nurse will be in charge of changing the wound dressing and will not be involved further in the trial. If unscheduled wound dressing is necessary, the indication and time point will be documented. In the case of wound complications, patients and investigators will be unblinded. The surgical report and all patient files will be blinded for surgical technique. To evaluate the success of the blinding procedure, the patient's and investigator's suspicion of group affiliation will be documented before measuring the primary endpoint and before regular unblinding at discharge. In the case of adverse events, unblinding may be performed prematurely based on the assessment of the principal investigator. The patients' allocated interventions can be revealed at any time by accessing the allocation list within the trials office.

### Interventions

#### Experimental intervention

Transvaginal hybrid NOTES sigmoid resection will be performed in the lithotomy position. A 5-mm trocar will be placed umbilically via an incision and a 12-mmHg carbon dioxide pneumoperitoneum established. Three additional 5-mm trocars will be placed under laparoscopic view: one suprapubically, one in the right lower quadrant, and one in the left lower quadrant (Figure 2). Before every skin incision, a local anesthetic (3 mL of bupivacaine 0.25%) will be applied subcutaneously. After laparoscopic exploration of the abdomen, standard medial-to-lateral mobilization of the sigmoid colon will be performed. For introduction of the stapler device, a 12-mm transvaginal trocar will be inserted through the posterior fornix after antiseptic wash-out of the vagina. After distal dissection of the sigmoid colon, a dorsal colpotomy will be performed and the colon carefully externalized through a transvaginal wound protector. Proximal dissection of the sigmoid colon will be performed extracorporally using an energy device. A purse-string suture will be placed at the proximal end of the colon and the circular stapling anvil inserted into the colon. After intra-abdominal relocation of the descending colon, the colpotomy will be sutured transvaginally with resorbable thread. An end-to-end anastomosis will be performed under laparoscopic view using a circular stapling device. Air-leak testing will be performed routinely by transanal insufflation of air with the anastomosis immersed in saline solution. After suturing the fascia and cutis, the standardized wound dressing will be placed.

#### Control intervention

Conventionally assisted laparoscopic sigmoid resection will be performed in the lithotomy position. A 5-mm umbilical trocar will be inserted via an incision. After establishing a 12-mmHg carbon dioxide pneumoperitoneum, three additional transabdominal trocars will be placed under laparoscopic view: one 12-mm trocar in the right lower quadrant, one 5-mm trocar in the left lower quadrant, and one 5-mm trocar at the suprapubic midline (Figure 3). Before every skin incision, local anesthetic (3 mL of bupivacaine 0.25%) will be applied subcutaneously. After medial-to-lateral mobilization of the descending colon, a laparoscopic stapling device will be used to dissect the distal end of the sigmoid colon. Pfannenstiel minilaparotomy will be performed to extract the sigmoid colon, extending the incision for the trocar placed within the suprapubic midline. After extracorporeal dissection of the proximal end, the anvil for the circular stapling device will be placed within the colon and secured with a purse-string suture. For colorectal anastomosis, the colon will be reintroduced into the peritoneal cavity. An end-to-end stapler anastomosis and air-leak test will be performed as described above. After suturing the fascia and cutis, the standardized wound dressing will be placed.

### Perioperative management, discharge, and follow-up

Perioperative thrombosis prophylaxis will be performed according to current German guidelines [13]. Single-shot antibiotics (ampicillin/sulbactam) will be given 30 minutes before surgery. Intraoperative anesthesia will comprise intravenous administration of piritramide. Postoperatively, pain medication will be administered according to the World Health Organization (WHO) analgesic ladder based on the judgment of the treating ward physician beginning directly after surgery in the recovery room [14]. Nonsteroidal anti-inflammatory drugs (metamizol) will be administered solely or in combination with a weak (tilidine) or strong opioid (piritramide, oxycodone). Peridural analgesia is not part of the pain management in this study. On the day of surgery and POD 1, analgesics will be administered intravenously. Beginning from POD 2, analgesics will be applied orally in cases of tolerated oral intake. Daily blood samples are scheduled for POD 1 to 5. Patients will be encouraged for discharge from POD 5 onward upon complete fulfillment of the discharge criteria (see ‘Discharge criteria’). Patients who receive the experimental intervention will be recommended to attend an ambulatory gynecological checkup 10 days after surgery. All patients will be followed up at an ambulatory checkup scheduled 3, 12, and 36 months after surgery. The ambulatory checkup will comprise taking a history, a clinical examination, and an assessment of secondary endpoints. Patients will be notified in writing and by phone in case they fail to return for their follow-up visits. If reaching the patients directly is not possible, their general practitioner will be contacted. Missing follow-up data will then be completed by phone.

### Discharge criteria

Patients will be discharged upon complete fulfillment of the following criteria:

-  ≥POD 5
-  Full oral intake
-  ≥1 passage of stool
-  Regular primary wound healing
-  Adequate pain control (VAS ≤3 under mobilization) with NSAID and low-potent opioid (tilidine)
-  In-ear body temperature ≤38°C
-  Decreasing laboratory markers of inflammation (CRP, leukocytes)
-  CRP, C-reactive protein; NSAID, nonsteroidal anti-inflammatory drug; POD, postoperative day; VAS, visual analogue scale.

### Risk-benefit ratio

The potential benefits of transvaginal hybrid NOTES sigmoid resection are expected to be less postoperative pain, faster convalescence, and reduced risk of wound infections and incisional hernia compared to conventional methods. The potential complications related to NOTES access are disturbed vaginal wound healing, impaired sexual function, and transvaginal microbiological contamination of the peritoneal cavity. In a recent study, we found a positive bacterial culture in the Douglas pouch following colpotomy in 2 of 27 (7%) patients undergoing transvaginal cholecystectomy. However, in both cases peritoneal contamination did not impact morbidity [15]. Two studies assessed postoperative sexual function after transvaginal NOTES cholecystectomy and did not find impairment after a follow-up of 12 months [16, 17]. In a cohort study of 44 patients undergoing transvaginal hybrid NOTES sigmoid resection we found major complications (≥grade 3) in 2 patients (4.4%) and minor complications (<grade 3) in 10 patients (22.2%) [11]. No mortality occurred [11]. Overall, these rates are comparable to previously published literature on assisted laparoscopic sigmoid resection for diverticular disease [18, 19]. Consequently, no evidence yet indicates higher morbidity after transvaginal hybrid NOTES sigmoid resection compared to the assisted laparoscopic approach.

### Outcome parameters

#### Primary endpoint

The primary endpoint is intensity of pain measured by a visual analogue scale (VAS) during mobilization of the patient 24 hours postoperatively. Patients and investigators will be blinded for this measurement. Before mobilization of the patient, the investigator will provide a standardized explanation of the VAS using a written text. The patient will be asked to record current pain intensity by drawing a vertical line on a horizontally positioned 100-mm VAS ranging from 0 (no pain) to 10 (worst imaginable pain). For mobilization, patients will be asked to take a seated position at the edge of the bed, to stand up, and finally to lie down in bed again. Immediately after mobilization, the patient will be asked to record the maximum pain intensity experienced during mobilization.

#### Secondary endpoints

Secondary endpoints will be measured intraoperatively, at daily study visits, and 3, 12, and 36 months postoperatively. Intraoperative parameters comprise operation time, length of minilaparotomy, height of the anastomosis and the length of the unfixed specimen. The number of harvested lymph nodes will be determined postoperatively by pathological examination. The schedule for study visits and follow-up is provided in Table 2. Pain intensity will be measured at rest and during mobilization in the same way as the primary endpoint. The dose and identity of applied analgesics will be recorded daily. Daily analgesic use will be graded using a numeric scale from one to three according to the WHO analgesic ladder [14]. Cumulative analgesic use will be assessed by adding daily WHO analgesic ladder scores. Daily patient mobility will be measured in meters by an electronic pedometer device, which will be placed on the wrist of the nondominant hand of the patient. Cumulative patient mobility will be assessed by adding daily distances. Inflammatory parameters (leukocytes, C-reactive protein) will be measured daily from POD 1 to POD 5. The discharge criteria (See ‘Discharge criteria’) will be evaluated beginning from POD 5. After discharge, the patients will be asked to record daily analgesic use and activity on a scale ranging from 0 (inactivity) to 5 (return to usual activities) until they return to usual activities and stop pain medication on a standardized form, which will be evaluated at the 3-month follow-up visit. Morbidity according to Dindo *et al*. [20] will be assessed at discharge, 3, 12, and 36 months postoperatively. The following patient-reported outcomes will be assessed preoperatively and 3, 12, and 36 months postoperatively: quality of life measured by the Gastrointestinal Quality of Life Index (GIQLI) [21], sexual function measured by the Female Sexual Function Index (FSFI) [22], and cosmetic satisfaction measured by the Body Image Scale [23].

**Table 2:** **Postoperative outcome parameters and schedule of study visits and follow-up**

Outcome parameter	Daily in-hospital study visits				Follow-up		
	POD 1 (24h) ^*^	POD 2 to POD 5	≥ POD6	Discharge	3 months	12 months	36 months
Pain intensity (VAS)	X (primary outcome)	X	X	X			
Daily analgesic use	X	X					
Cumulative analgesic use		X (POD 5)^†^		X	X		
Daily patient mobility	X	X		X			
Cumulative patient mobility				X			
Inflammatory parameters (CRP, leukocytes)	X	X					
Discharge criteria		X (POD5)^†^	X				
Length of stay				X			
Morbidity				X	X	X	X
Return to normal activities					X		
Quality of life (GIQLI [21])					X	X	X
Sexual function (FSFI [22])					X	X	X
Cosmetic satisfaction (Body Image Scale [23])					X	X	X

^*^Study visit on POD 1 is scheduled 24 hours postoperatively; ^†^parameters will be measured solely on POD 5. CRP, C-reactive protein; FSFI, Female Sexual Function Index; GIQLI, Gastrointestinal Quality Of Life Index; POD, postoperative day; VAS, visual analogue scale.

### Data management

Data will be entered in a CRF by the investigator or a designated representative of the respective center. Participant names and collected data are subject to medical confidentiality. In the case of resignation, collected data may be pseudonymized unless the participant explicitly requests that all data be erased. Data will be entered into the CRF as soon as possible after data retrieval. After completion, the original CRF will be sent to the SDGC for validation and transfer into the trial database. Double data entry will be performed in order to ensure accurate transfer from the CRF to the database. At the end of the trial, the original CRFs and final database will be archived by the principal investigator, who is responsible for providing data to trial investigators. All trial members are obliged to ensure the deletion of duplicate data recordings at the end of the trial.

### Safety and reporting of serious adverse events

Serious adverse events (SAEs), defined according to the guidelines for good clinical practice by the International Conference on Harmonization of Technical Requirements for Registration of Pharmaceuticals for Human Use (ICH-GCP), will be reported from the day of first enrollment until the regular end of the trial [24]. All SAEs will be documented in a separate ‘serious adverse event form’ and the CRF, and will be reported to the principal investigator within 24 hours of being noted. If the principal investigator considers a SAE as unexpected and related to the study intervention, he will submit a report to the local ethics committee within 3 days. Furthermore, morbidity will be documented within the CRF. The study steering committee will meet to evaluate morbidity and SAEs at least twice: after randomization of one-third of the patients and after randomization of two-thirds of the patients. In the case of relevant imbalances between the groups, a report will be submitted to the local ethics committee. The trial may be terminated based on the decision of the principal investigator according to the assessment of the local ethics committee.

### Statistical methods

#### Sample size

Sample size was determined for the primary endpoint, the intensity of pain during mobilization measured on the VAS 24 hours after surgery. Park *et al*. reported a mean (± standard deviation) VAS score of 4.2 ± 1.7 for transvaginal hybrid NOTES vs. 5.7 ± 1.7 for conventional laparoscopic right-sided hemicolectomy [10]. According to Gallagher *et al*. a standardized effect size of unity in the VAS score (difference in unity given a standard deviation of unity) is discriminable by patients and can be considered clinically relevant [25]. We made the following assumptions when calculating the patient number:

-  Hypothesis: transvaginal hybrid NOTES sigmoid resection causes less pain than laparoscopic-assisted sigmoid resection 24 hours after surgery (superiority analysis).
-  Two-sided analysis with a type I error (α) of 0.05 and power (1-β) of 0.90. The standardized effect size was assumed to be 1 (difference in VAS of 1 and standard deviation of 1). The power was set at 0.90 because some evidence is already available.

Based on these assumptions, a sample size of 23 patients per arm was calculated using the R package (version 0.1-8; http://cran.r-project.org/web/packages/Sample.Size/index.html, *t* test for two independent samples). Assuming a drop-out rate of 20%, the total number of patients needed per arm is 29, resulting in a total patient number of 58 patients for the primary arm.

#### Statistical analysis

Statistical analysis will be performed using the R statistical software ( http://www.r-project.org). A two-sided *P* value <0.05 will be considered significant. For baseline characteristics, descriptive statistics will be used. For analysis of the primary outcome, a Mann-Whitney *U* test will be applied. For categorical secondary endpoints, chi-square statistics will be used. To compare continuous secondary endpoints (operating time, length of hospital stay, analgesic use, time to return to normal activity), Mann-Whitney *U* tests will be applied. No interim analysis is planned for this study. For the time course of pain at provocation and at rest, a mixed model with mean VAS scores will be applied.

Missing values will be replaced with the last available value (the last observation carried forward (LOCF) approach). Data from patients who withdraw from the study will be disregarded unless exclusion is based on postoperative patient wishes and the patient agrees to the use of the already obtained data.

The confirmatory analysis will be performed based on intention-to-treat (ITT) patients and with respect to ITT principles. A standard sensitivity analysis will be performed on the per-protocol population.

### Ethical approval

The ethics committee of the University of Heidelberg reviewed and approved this study on January 13, 2014 (reference number: S-608/2013).

### Good clinical practice

The trial was conceived and will be conducted according to all relevant national and international rules and regulations, such as the guidelines for good clinical practice by the ICH-GCP [24] and Declaration of Helsinki (2013) [26].

### Dissemination policy

The results of the TRANSVERSAL trial are intended to be presented at international medical congresses on corresponding fields of interest, for example, general and visceral surgery or endoscopic surgery. Written publications are planned within surgical or endoscopic scientific journals. The authorship for written publications has to be confirmed unequivocally by all lead investigators and will only be granted in the case of substantive contributions to the design, conduct, data analysis, and interpretation. The contact data for all lead investigators and access to the full study protocol will be presented online on the website of the surgical department of the University of Heidelberg ( http://www.klinikum.uni-heidelberg.de/Chirurgische-Klinik.1010.0.html). After completion of the full study report, anonymized participant-level datasets and the statistical code for generating results will be available by contacting the principal investigator.

### Registration

The study protocol has been registered with the German Clinical Trials Register ( https://drks-neu.uniklinik-freiburg.de/drks_web/) under the registration number DRKS00005995.

### Protocol version

This manuscript refers to the fourth version of the full study protocol issued on August 26, 2014. Protocol modifications will be reported to all investigators, the local ethics committee, the German Clinical Trials Register, all trial participants, and the journal.

## Discussion

NOTES represents a consequence of developing minimally invasive surgery to reduce abdominal wall trauma using natural orifice access to the peritoneal cavity. Laparoscopic-assisted surgery may eventually advance to pure laparoscopic surgery, as minilaparotomy can be avoided by using the natural orifice for specimen retrieval. Since the white paper in which members of the Society of American Gastrointestinal and Endoscopic Surgeons (SAGES) and American Society for Gastrointestinal Endoscopy (ASGE) presented future requirements for the development and clinical application of NOTES, clinical and experimental studies have focused on feasibility [27]. Although transvaginal NOTES procedures have already entered the clinical routine, only a few studies have compared NOTES to current standard surgical treatment [10, 28, 29].

Pain intensity 24 hours after surgery has been chosen as a primary endpoint of TRANSVERSAL trial because pain is one of multiple outcomes reflecting trauma and recovery after abdominal surgery [30]. In contrast to other factors, pain may be objectified well using the VAS, a test with high validity [31]. Finally, because pain affects a variety of physiological processes, such as the stress response, inflammation, and wound healing, we think that pain may represent a decisive factor within the recovery process. We decided to measure pain intensity during mobilization of the patient because, even under closely monitored analgesic treatment, pain may be provoked by mobilization and affect postoperative patient mobility. The secondary endpoints of the TRANSVERSAL trial comprise additional outcomes reflecting recovery, such as postoperative patient mobility measured by an electronic pedometer, quality of life, length of hospital stay, and time to return to normal activity.

To the best of our knowledge only three randomized controlled trials have compared transvaginal hybrid NOTES to laparoscopic surgery, and all of the studies assessed the transvaginal NOTES technique applied to cholecystectomy [28, 29, 32]. In 60 female patients, Noguera *et al.* found no differences with regard to morbidity, postoperative pain, length of stay, and time off work for hybrid transvaginal NOTES, hybrid transumbilical NOTES, and conventional laparoscopic cholecystectomy at 1-year follow-up. In contrast, in a recent randomized controlled trial including 40 female patients, Bulian *et al*. found that the transvaginal hybrid NOTES technique is associated with less postoperative pain, less analgesic use, and improved postoperative quality of life compared to needlescopic three-trocar cholecystectomy in the short term (POD 1 to POD 10). Borchert *et al*. found no significant difference between transvaginal hybrid NOTES and laparoscopic cholecystectomy with regard to postoperative pain and safety in 97 patients in the short term (POD 1 to POD 7).

With regard to colorectal surgery, clinical evidence for NOTES procedures is limited to cohort studies and one matched controlled study. Cohort studies have shown the feasibility of transvaginal and transanal specimen extraction for colonic resection [11, 33–35]. In a matched cohort study including 68 patients, Park *et al*. found that transvaginal specimen extraction for right-sided hemicolectomy is associated with less pain and shorter hospital stay than the laparoscopic-assisted technique. No difference was found with regard to morbidity after a median follow-up of 23 months [10].

Despite increasing clinical application, NOTES has still encountered broad skepticism because its potential benefits are generally doubted and potential access-related complications feared. The TRANSVERSAL trial is the first study comparing NOTES to conventionally assisted surgery for colonic resection in a randomized controlled setting. The results of this trial will provide valuable clinical evidence for objective assessment of the potential benefits and risks of NOTES procedures compared to the current surgical standard.

## Trial status

Recruitment will begin in the fourth quarter of 2014.

## Electronic supplementary material

Additional file 1: **WHO Trial Registration Data Set.** (DOCX 120 KB)

Additional file 2: **Patient Information Sheet.** (PDF 93 KB)

Additional file 3: **Consent Form.** (PDF 60 KB)

Additional file 4: **Standardized Follow Up Form.** (DOCX 56 KB)

## Abbreviations

CRF: case report form
ICH-GCP: guidelines for good clinical practice constituted by the International Conference on Harmonization of Technical Requirements for Registration of Pharmaceuticals for Human Use
ITT: intention-to-treat
NOTES: natural orifice transluminal endoscopic surgery
POD: postoperative day
SAE: serious adverse event
VAS: visual analogue scale.
