# Supplementary material for: The transvaginal hybrid NOTES versus conventionally assisted laparoscopic sigmoid resection for diverticular disease (TRANSVERSAL) trial: study protocol for a randomized controlled trial
Source: Trials. 2014 Nov 20;15:454. doi: 10.1186/1745-6215-15-454 (PMC4246541; doi:10.1186/1745-6215-15-454)
Supplement: Supplementary file 1 — Additional file 1: WHO Trial Registration Data Set. (DOCX 120 KB) [file 13063_2014_2309_MOESM1_ESM.docx]

| **Data category** | **Information** |
| --- | --- |
| Primary registry and trial identifying number | German Clinical Trials Register (<https://drks-neu.uniklinik-freiburg.de/drks_web/>), registration number: DRKS00005995 |
| Date of registration in primary registry | 27.03.2014 |
| Secondary identifying numbers | n/a |
| Source(s) of monetary or material support | Institutional means of the University of Heidelberg |
| Primary sponsor | University of Heidelberg |
| Secondary sponsor(s) | n/a |
| Contact for public queries | PD Dr. med. Georg Linke  Department of General, Abdominal and Transplantation Surgery  University of Heidelberg  Im Neuenheimer Feld 110  69120 Heidelberg, Germany  phone: ++49 (0) 6221 56 8641  fax: ++49 (0) 6221 56 8645 |
| Contact for scientific queries | PD Dr. med. Georg Linke  Department of General, Abdominal and Transplantation Surgery  University of Heidelberg  Im Neuenheimer Feld 110  69120 Heidelberg, Germany  phone: ++49 (0) 6221 56 8641  fax: ++49 (0) 6221 56 8645 |
| Public title | Transvaginal hybrid NOTES versus conventionally assisted laparoscopic sigmoid resection |
| Scientific title | The TRANSVERSAL trial: Transvaginal hybrid NOTES versus conventionally assisted laparoscopic sigmoid resection for diverticular disease - a randomized controlled trial |
| Countries of recruitment | Germany |
| Health condition(s) or problem(s) studied | Diverticular disease of the sigmoid colon |
| Intervention(s) | Arm 1: Transvaginal hybrid NOTES sigmoid resection  Arm 2: Laparoscopic assisted sigmoid resection |
| Key inclusion and exclusion criteria | Ages eligible for study: ≥18 years  Sexes eligible for study: female  Inclusion criteria:  Elective surgical indication for sigmoid resection due to complicated or reoccurring sigmoid diverticulitis classified as IIa, IIb or III according to Hansen and Stock; informed consent;  Exclusion criteria:  ASA classification higher than III, pregnancy, Genital infections, Neoplasms of vulva, vagina or cervix, Douglas endometriosis, History of pelvic floor repair, Chronic inflammatory bowel disease, Fibromyalgia, Psychiatric disorder, Regular use of analgetics, steroids or anti-depressants |
| Study type | Interventional  Allocation: randomized  Intervention model: parallel assignment  Masking: double blind (subject, caregiver, investigator, outcomes assessor)  Primary purpose: therapy |
| Date of first enrolment | n/a |
| Target sample size | 58 |
| Recruitment status | Recruitment is planned for the 4^th^ quarter of 2014. |
| Primary outcome(s) | Intensity of pain measured by a Visual Analogue Scale (VAS) during mobilization of the patient 24 hours postoperatively |
| Key secondary outcomes | Postoperative patient mobility, daily pain intensity, analgesic use, operation time, length of mini-laparotomy, intraoperative complications, time until first stool passage, inflammatory parameters (leucocytes, C-reactive protein), duration of hospital stay, return to normal activity, morbidity, quality of life, sexual function and cosmetic satisfaction. |
