# Supplementary material for: The transvaginal hybrid NOTES versus conventionally assisted laparoscopic sigmoid resection for diverticular disease (TRANSVERSAL) trial: study protocol for a randomized controlled trial
Source: Trials. 2014 Nov 20;15:454. doi: 10.1186/1745-6215-15-454 (PMC4246541; doi:10.1186/1745-6215-15-454)
Supplement: Supplementary file 2 — Additional file 2: Patient Information Sheet. (PDF 93 KB) [file 13063_2014_2309_MOESM2_ESM.pdf]

Patientinneninformation

Transvaginale rigid-hybrid NOTES Sigmaresektion
# versus
laparoskopisch assistierte Sigmaresektion

-

Eine randomisiert kontrollierte Studie

(TRANSVERSAL- Studie)

Sehr geehrte Patientin,

sie leiden unter einer Erkrankung Ihres s-förmigen Dickdarms (Sigma) im linken Unterbauch und es wurde Ihnen empfohlen, den erkrankten Darmabschnitt operativ entfernen zu lassen.

Dieser Eingriff wird heute normalerweise als laparoskopische Operation, das heißt als „Schlüsselloch-Eingriff" durchgeführt. Dabei werden über vier kleine Bauchschnitte eine Kamera und Instrumente in den Bauch eingeführt. Mit diesen Instrumenten wird unter Videosicht der erkrankte Darmabschnitt mobilisiert und dann über einen ca. 7-8 cm langen Bauchschnitt im Bereich des Unterbauchs geborgen.

Zurzeit überprüfen wir in einer Studie die Praktikabilität einer neuen Operationstechnik, der sogenannten NOTES-Technik (**N**atural **O**rifice **T**ransluminal **E**ndoscopic **S**urgery). Bei der NOTES-Technik werden nicht mehr alle, sondern nur noch ein Teil der Instrumente über Bauchschnitte eingeführt. Einige Instrumente (z.B. Kamera, Fasszange und Schere) werden dabei über die Scheide in den Bauchraum eingebracht. Über diesen Zugang durch die Scheide kann dann am Ende der Operation der entfernte Darmabschnitt aus dem Bauchraum geborgen werden. Im Gegensatz zur herkömmlichen laparoskopisch assistierten Operation ist bei dieser neuen Methode kein größerer Bauchschnitt mehr nötig. Möglicherweise kommt es bei dieser Operationstechnik zu weniger Schmerzen und seltener zu Wundinfekten oder Narbenbrüchen im Bauchbereich kommt. Außerdem haben Sie weniger Narben auf Ihrem Bauch, also ein verbessertes kosmetisches Ergebnis.

Ziel:

Durch zufällige Anwendung der einen oder anderen oben beschriebenen Operationstechnik soll überprüft werden, ob das neue Operationsverfahren tatsächlich weniger operationsbedingte Schmerzen, Wundinfekte oder Narbenbrüche aufweist. Welches der beiden Verfahren überlegen ist, ist momentan nicht erwiesen. Diese Frage soll mit der vorliegenden Studie untersucht werden. Die Zuordnung der Patientinnen zu dem jeweiligen Verfahren erfolgt hierbei nach dem Zufallsprinzip (einem Verteilungsschema, das vor

Untersuchungsbeginn festgelegt wird). Dies hat den Zweck, eine möglichst hohe wissenschaftliche Aussagekraft der Untersuchung zu erreichen. Die zufällige Zuordung erfolgt während der Operation nach Durchführung einer Bauchspiegelung und Vergewisserung der technischen Durchführbarkeit beider Verfahren.

Vor- und Nachteile der Studienteilnahme:

Bei einer NOTES Sigmaresektion werden größere Bauchschnitte zur Bergung des zu entfernenden Darmabschnittes vermieden. Hierdurch sollten im Vergleich zur laparoskopisch assistierten Technik nach der Operation weniger Schmerzen auftreten und es sollte seltener zu Wundinfekten oder Narbenbrüchen im Bauchbereich kommen. Außerdem haben Sie weniger Narben auf Ihrem Bauch, also ein verbessertes kosmetisches Ergebnis.

Da sich die eingesetzte standardisierte operative Technik bis auf den Zugang nicht von der üblichen laparoskopisch assistierten Technik unterscheidet, ist eine im Vergleich zur klassischen laparoskopisch assistierten Operation vergleichbare Komplikationsrate zu erwarten. In einer Voruntersuchung an 45 Patienten zeigte sich eine Gesamtkomplikationsrate von 26,7% (12/45). Schwerwiegende Komplikationen traten mit einer Häufigkeit von 4,4% (2/45) auf.

Neben den bekannten Risiken einer laparoskopisch assistierten Sigmaresektion bestehen beim Zugang über die Scheide Risiken bezüglich der vaginalen Wundheilung. Ebenso wie im Bereich der Bauchwandwunden kann es im Bereich der Scheide zu einer Wundheilungsstörung kommen. Das zugangsspezifische Risiko der transvaginalen Laparoskopie entspricht voraussichtlich dem Risiko der transvaginalen Bauchspiegelung in der Gynäkologie, ein Verfahren, das standardmäßig in der Gynäkologie eingesetzt wird und als komplikationsarm gilt. Komplikationen im Bereich der Vagina traten in der genannten Studie mit einer Häufigkeit von 6,6% (3/45) auf. Hierbei handelte sich es allerdings um geringfügige Komplikationen, die keine erneute Krankenhausbehandlung oder Operation zur Folge hatten. Als seltene Spätfolge kann es zu Schmerzen (auch beim Geschlechtsverkehr) im Bereich der Vagina kommen. Untersuchungen nach transvaginalen Gallenblasenoperationen konnten bisher allerdings keine relevante Beeinträchtigung der Sexualfunktion nachweisen. Ein weiteres Risiko ist durch eine theoretisch mögliche Verschleppung vaginaler Keime in die Bauchhöhle gegeben. Allerdings ergab sich in den bisherigen Untersuchungen kein Anhalt für ein erhöhtes Infektionsrisiko nach transvaginalen Eingriffen.

Verblindung:

Um die beiden zu vergleichenden Verfahren objektiv zu untersuchen werden die Patientinnen, Untersucher und das Pflegepersonal gegenüber dem angewendeten Operationsverfahren zunächst verblindet. Die Verblindung wird durch Anlage eines sterilen Wundverbandes im Bereich des Unterbauchs realisiert. Dieser Verband wird am 2. postoperativen Tag und

danach in 3-tägigen Abständen gewechselt. Um die Verblindung aufrecht zu erhalten ist es notwendig, dass Sie im Rahmen der Verbandswechsel kurzeitig eine Augenblende tragen. Bei Entlassung aus der stationären Versorgung wird der Wundverband endgültig entfernt und Sie werden über das bei Ihnen durchgeführte Verfahren informiert.

Datenerhebung und Datenschutz:

Im Rahmen dieser Studie sollen Daten erhoben werden, die dem Vergleich der transvaginalen NOTES Sigmaresektion mit der laparoskopisch assistierten Sigmaresektion dienen.

Gemessen werden das Ausmaß der postoperativen Schmerzen, der Schmerzmittelbedarf, die Mobilität nach Operation, die Operationszeit, die Länge des entfernten Dickdarmpräparats und die Anzahl der darin enthaltenen Lymphknoten, die Häufigkeit von Komplikationen, Laborwerte (Entzündungsparameter), Krankenhausverweildauer und Rekonvaleszenzzeit. Die Datenerhebung erfolgt vor der Operation, im Rahmen von täglichen Visiten während des stationären Aufenthalts und zu den jeweiligen Nachuntersuchungsterminen (3, 12 und 36 Monate nach Operation). Die Messung der Mobilität nach Operation erfolgt durch einen handelsüblichen elektronischen Schrittzähler (Pedometer). Zur Messung der Lebensqualität, der Sexualfunktion und des kosmetischen Ergebnisses müssen vor Operation und bei den Nachuntersuchungen jeweils drei Fragebögen ausgefüllt werden. Die Bearbeitung eines Fragebogens nimmt 5-10 Minuten in Anspruch. Bei Entlassung aus der stationären Versorgungen wird Ihnen ein Tagebuch ausgehändigt, das der Dokumentation der Einnahme von Schmerzmedikamenten und der Alltagsfähigkeit dient. Die tägliche Dokumentation sollte nicht mehr als 3-5 Minuten in Anspruch nehmen.

Alle Daten zu dieser Studie werden in einem gesonderten Dokumentationsbogen erhoben und pseudonymisiert d.h. es werden keine Angaben von Namen oder Initialen verwendet. Es werden, ausschließlich Nummern- und/oder Buchstabencodes, evtl. mit Angabe des Geburtsjahres, statistisch ausgewertet und ggf. in dieser Form weitergegeben. Dritte erhalten keinen Einblick in Originalunterlagen. Zur Überprüfung und Vervollständigung dieser Daten ist gelegentlich ein Vergleich mit Ihrer Patientenakte oder der Austausch mit Ihrem Hausarzt, oder einem anderen der Sie behandelnden Ärzte erforderlich. Mit Ihrer Studienteilnahme erklären Sie Ihr Einverständnis, dass der Hausarzt und andere Sie behandelnde Ärzte diesbezüglich gegenüber den Studienverantwortlichen von der ärztlichen Schweigepflicht entbunden werden. Dabei werden strenge Vertraulichkeit gewahrt und die Grundsätze der ärztlichen Schweigepflicht sowie des Bundesdatenschutzgesetzes eingehalten.

Freiwilligkeit, vorzeitige Beendigung der Teilnahme:

Die Teilnahme an der Studie ist für Sie vollkommen freiwillig. Sie können jederzeit ohne Angaben von Gründen Ihre Zustimmung zur Studienteilnahme widerrufen, ohne irgendeinen Nachteil für Ihre weitere medizinische Versorgung befürchten zu müssen. Bei Rücktritt von

der Studie können, falls Sie dies wünschen, bereits gewonnene Daten gelöscht werden. Ihre Studienstammdaten werden bis zum Abschluss der Untersuchungen und der Datenauswertung im Studienzentrum aufbewahrt. Es ist eine Gesamtstudiendauer von 60 Monaten vorgesehen. Die Untersuchung wird in Übereinstimmung mit der ärztlichen Berufsordnung durchgeführt. Sie können an weiteren Studien teilnehmen, wenn keine Überschneidung mit dieser Studie vorliegt. Hierzu können Sie sich jederzeit mit Ihrem Studienarzt besprechen

Sollten Sie noch weitere Fragen zur Studie haben, zögern Sie bitte nicht, diese uns bzw. Ihrem behandelnden Arzt zu stellen. Gerne versuchen wir, Ihnen Ihre Fragen so ausführlich und so umfassend wie möglich zu beantworten.

Für Rückfragen stehen Ihnen folgende Personen zur Verfügung:

Studienverantwortlicher und Studienleiter

Name	PD Dr.med. Beat P. Müller
Abteilung	Klinik für Allgemein-, Viszeral-, und Transplantationschirurgie<br>Universitätsklinikum Heidelberg<br>Im Neuenheimer Feld 110<br>D-69120 Heidelberg
Telefon	+49 6221 56 4828
Fax	+49 6221 56 86 45
E-Mail	beat.mueller@med.uni-heidelberg.de

Studienkoordinator

Name	Dr. med. Georg R. Linke
Abteilung	Klinik für Allgemein-, Viszeral-, und Transplantationschirurgie<br>Universitätsklinikum Heidelberg<br>Im Neuenheimer Feld 110<br>D-69120 Heidelberg
Telefon	+49 6221 56 36087
Fax	+49 6221 56 86 45
E-Mail	georg.linke@med.uni-heidelberg.de
