# Supplementary material for: The transvaginal hybrid NOTES versus conventionally assisted laparoscopic sigmoid resection for diverticular disease (TRANSVERSAL) trial: study protocol for a randomized controlled trial
Source: Trials. 2014 Nov 20;15:454. doi: 10.1186/1745-6215-15-454 (PMC4246541; doi:10.1186/1745-6215-15-454)
Supplement: Supplementary file 3 — Additional file 3: Consent Form. (PDF 60 KB) [file 13063_2014_2309_MOESM3_ESM.pdf]

Einwilligungserklärung

Transvaginale rigid-hybrid NOTES Sigmaresektion
# versus
laparoskopisch assistierte Sigmaresektion

-

Eine randomisiert kontrollierte Studie

(TRANSVERSAL - Studie)

Die schriftliche Patientinneninformation zur **TRANSVERSAL - Studie** habe ich erhalten und gelesen. Darüber hinaus bin ich durch Herrn / Frau ______________________ mündlich aufgeklärt worden. Dabei wurden alle meine Fragen beantwortet.

Ich stimme der Teilnahme an der Studie freiwillig zu. Ich weiß, dass ich meine Zustimmung jederzeit und ohne Begründung und ohne Nachteile für meine weitere medizinische Versorgung widerrufen kann. Bei Rücktritt von der Studie bin ich mit der Auswertung der schon gewonnenen Untersuchungsdaten einverstanden/nicht einverstanden (bitte zutreffendes unterstreichen).

Ich wurde darüber aufgeklärt und stimme zu/nicht zu (bitte zutreffendes unterstreichen), dass meine in der Studie erhobenen Daten in pseudonymisierter Form aufgezeichnet und ggf. auch in pseudonymisierter Form weitergegeben werden können. Ich bin einverstanden, dass Krankendaten, die diese Studie betreffen, mit meinem Hausarzt ausgetauscht werden können. Dritte erhalten keinen Einblick in Originalkrankenunterlagen. Bei der Veröffentlichung der Ergebnisse dieser Studie wird mein Name ebenfalls nicht genannt.

______________________________	______________________________
Vor-/Nachname der Patientin	Geburtsdatum der Patientin
______________________________	______________________________
Aufklärungsdatum<br>(von der Patientin einzutragen)	Unterschrift der Patientin
______________________________	______________________________
Aufklärungsdatum<br>(vom Prüfer einzutragen)	Unterschrift des Prüfers
