# Supplementary material for: The transvaginal hybrid NOTES versus conventionally assisted laparoscopic sigmoid resection for diverticular disease (TRANSVERSAL) trial: study protocol for a randomized controlled trial
Source: Trials. 2014 Nov 20;15:454. doi: 10.1186/1745-6215-15-454 (PMC4246541; doi:10.1186/1745-6215-15-454)
Supplement: Supplementary file 4 — Additional file 4: Standardized Follow Up Form. (DOCX 56 KB) [file 13063_2014_2309_MOESM4_ESM.docx]

**TRANSVERSAL-Studie**

**Transvaginale rigid-hybrid NOTES Sigmaresektion**

**versus**

**laparoskopisch assistierte Sigmaresektion**

**Patiententagebuch**

Zentrumsnummer:

Screeningnummer:

Bitte tragen Sie täglich die eingenommene Schmerzmedikation und den Skalawert für Ihre Aktivität nach unten dargestelltem Schema in das Tagebuch ein. Wir bitten Sie das Tagebuch bis zum Zeitpunkt bis zum 30. postoperatvien Tag nach Operation, d.h. in Ihrem Fall bis zum _________________ zu führen und zum ersten Nachuntersuchungstermin (3 Monate nach Operation) mitzubringen.

**Schmerzmedikation:**

Bitte tragen Sie täglich die eingenommenen Schmerzmedikamente nach folgendem Schema ein:

Beispiel: Einnahme von Novalgin 500 mg nach Bedarf. Es wurde jeweils eine Tablette am Morgen und eine am Abend eingenommen:

Medikament: Novalgin

Dosis: 500 mg

Zeitpunkt der Einnahme: 1x morgens, 1 x abends

**Aktivitätsskala:**

Bitte markieren Sie täglich den zutreffenden Wert für die Aktivität:

1: Keine Aktivität: Bettlägerigkeit

2: Wenig Aktivität: gelegentliches Aufstehen aus dem Bett, Bewegung nur innerhalb des Wohnraumes.

3: Mäßige Aktivität: Ausübung von Alltagstätigkeiten unter Unterstützung durch weitere Personen (Z.B. Spazieren gehen, Kochen, Einkaufen)

4: Teilweise Alltagsfähigkeit: Teilweise selbstständige Ausübung von gewohnten Alltagstätigkeiten (Z.B. Spazieren gehen, Kochen, Einkaufen)

5: Vollständige Alltagsfähigkeit: Selbstständige Ausübung aller gewohnten Alltagstätigkeiten (Z.B. Spazieren gehen, Kochen, Einkaufen, Arbeit, Hobbies, Achtung: Sportliche Betätigung/Schweres Heben ausgenommen)

**Datum:**

**Schmerzmedikation:**

Medikament 1: Dosis: Einnahme:

Medikament 2: Dosis: Einnahme:

**Aktivitätskala (1-5):**

1: Keine Aktivität: Bettlägerigkeit 2: Wenig Aktivität: gelegentliches Aufstehen aus dem Bett, Bewegung nur innerhalb des Wohnraumes. 3: Mäßige Aktivität: Ausübung von Alltagstätigkeiten unter Unterstützung durch weitere Personen (Z.B. Spazieren gehen, Kochen, Einkaufen) 4: Teilweise Alltagsfähigkeit: Teilweise selbstständige Ausübung von gewohnten Alltagstätigkeiten (Z.B. Spazieren gehen, Kochen, Einkaufen) 5: Vollständige Alltagsfähigkeit: Selbstständige Ausübung aller gewohnten Alltagstätigkeiten (Z.B. Spazieren gehen, Kochen, Einkaufen, Arbeit, Hobbies, Achtung: Sportliche Betätigung/Schweres Heben ausgenommen)
